# In-Hospital 3D Printed Scaphoid Prosthesis Using Medical-Grade Polyetheretherketone (PEEK) Biomaterial

**DOI:** 10.1155/2021/1301028

**Published:** 2021-01-11

**Authors:** Philipp Honigmann, Neha Sharma, Ralf Schumacher, Jasmine Rueegg, Mathias Haefeli, Florian Thieringer

**Affiliations:** ^1^Hand Surgery, Cantonal Hospital Baselland, Rheinstrasse 26, 4410 Liestal, Switzerland; ^2^Medical Additive Manufacturing Research Group, Department of Biomedical Engineering, University of Basel, Gewerbestrasse 16, 4123 Allschwil, Switzerland; ^3^Amsterdam UMC, University of Amsterdam, Department of Biomedical Engineering and Physics, Amsterdam Movement Sciences, Meibergdreef 9, Amsterdam, Netherlands; ^4^Department of Oral and Cranio-Maxillofacial Surgery, University Hospital Basel, Spitalstrasse 21, 4031 Basel, Switzerland; ^5^Medartis AG, Hochbergerstrasse 60 E, 4057 Basel, Switzerland; ^6^Hand Surgery, Cantonal Hospital of Grisons, Loestrasse 170, 7000 Chur, Switzerland

## Abstract

Recently, three-dimensional (3D) printing has become increasingly popular in the medical sector for the production of anatomical biomodels, surgical guides, and prosthetics. With the availability of low-cost desktop 3D printers and affordable materials, the in-house or point-of-care manufacturing of biomodels and Class II medical devices has gained considerable attention in personalized medicine. Another projected development in medical 3D printing for personalized treatment is the in-house production of patient-specific implants (PSIs) for partial and total bone replacements made of medical-grade material such as polyetheretherketone (PEEK). We present the first in-hospital 3D printed scaphoid prosthesis using medical-grade PEEK with fused filament fabrication (FFF) 3D printing technology.

## 1. Introduction

Additive manufacturing, also known as three-dimensional (3D) printing, is a growing trend in the medical field. Even though 3D printing technology is over 30 years old. This aspect is distinctly evident with an exponential increase in the number of publications on 3D printing in the medical specialties, especially in the orthopedic field [1, 2].

**Figure 1:**
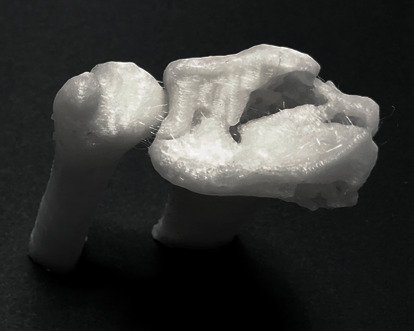
In-house printed fracture model using an FFF consumer-level desktop 3D printer (MakerBot Replicator+, MakerBot Brooklyn, New York City, New York, USA).

Medical 3D printing has entirely transformed the current era of personalized medicine with its state-of-art usefulness and applications. With the utilization of consumer-level desktop 3D printers in hospitals, 3D printing offers several medical and clinical applications including, but not limited to, anatomical, pathological fracture, and tumor biomodels, customized surgical tools, and prosthetic aids [3–8]. This technology can build a 3D object by creating complex, customized anatomical and medical structures as defined in a computer-aided design (CAD) digital file. In a basic technical setup, the two-dimensional (2D) Digital Imaging and Communications in Medicine (DICOM) medical imaging datasets are converted into 3D data, which are transferred to a 3D printer. An illustration of an in-house 3D printed biomodel for a distal intra-articular radius fracture case, fabricated via a fused filament fabrication (FFF) 3D printing technology using cone-beam computed tomography (CBCT) DICOM dataset, is shown in Figure 1 [9].

While the fabrication of biomodels is easily conceivable at the point-of-care, the production of Class II medical devices such as surgical guides that come in contact with the patient's blood was usually outsourced to external sources. These medical devices need to be printed with certified biocompatible materials requiring expensive professional certified 3D printers, which were not affordable to many hospitals, and therefore, these products were often printed externally by certified companies [2]. However, recently with the availability of in-house 3D printing setups and affordable desktop 3D printers, the fabrication of surgical guides has slowly shifted from external service providers to the hands of the clinicians. Another projected development in medical 3D printing for personalized treatment is the in-house fabrication of patient-specific implants (PSIs) such as osteosynthesis plates and prosthesis.

With a significant change from the old mass-production system of medical implants to the PSI production system, 3D printing has attained an essential place in the medical implant manufacturing industry. In consideration of the evolving technological trends in personalized medicine, we investigated the printing feasibility of medical-grade polyetheretherketone (PEEK) biomaterial, especially for the production of PSIs in a hospital environment. Our preliminary results were promising, which contributed towards the progression of an FFF PEEK 3D printer solely designed for medical PEEK applications [10]. Later in 2018, Honigmann et al. presented the first cadaveric results of a patented 3D printed titanium patient-specific scaphoid prosthesis [11]. This patient-specific prosthesis was designed for cases of nonreconstructable scaphoids because of nonunion or trauma.

More recently, the use of polymer as an alternative to metallic biomaterials is being explored. PEEK meets the perfect criteria for the orthopedic field as a printable material for PSIs [12]. It is a lightweight, biocompatible, nontoxic, and noninflammable biomaterial exhibiting excellent mechanical strength [13]. The osteoconductive properties of PEEK support the bone integration process [14]. Moreover, PEEK is radiolucent in X-ray imaging with no relevant artifacts, providing computed tomography (CT) and magnetic resonance imaging (MRI) compatibility. These inherent advantageous characteristics of PEEK, along with the capability to print medical-grade PEEK in a certified 3D printer, make this material an attractive option suitable for 3D printed PSIs at the hospital or point-of-care manufacturing [10]. Therefore, in this article, we present the preliminary results on the first in-house 3D printed scaphoid prosthesis made of medical-grade PEEK fabricated via material extrusion (FFF) 3D printing.

## 2. Materials and Methods

### 2.1. Computer-Aided Design Modeling of the Scaphoid Prosthesis

**Figure 2:**
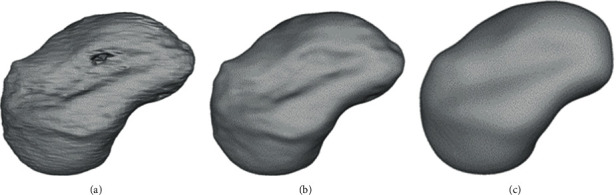
Surface smoothening of the scaphoid prosthesis: (a) native; (b) filled holes; (c) final smoothened surface.

**Figure 3:**
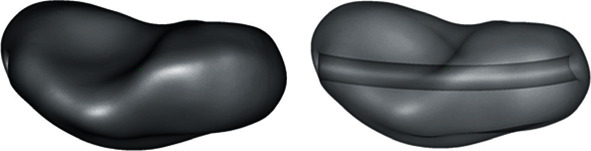
Design of the curved channel in the scaphoid prosthesis.

The anatomical department provided a Thiel conserved wrist with no degenerative changes or posttraumatic changes. A multislice CT scan (Biograph mCT Flow™, Siemens Medical Solutions USA Inc., Malvern, USA) was used to acquire the DICOM dataset. The DICOM files were processed in a medically certified image processing software (Mimics®, Materialise, Leuven, Belgium) to generate a 3D volumetric reconstruction model of the scaphoid. The native surface of the generated 3D model was smoothened, and mesh repairing procedures such as fixing holes were executed in a CAD software (3DS Geomagic Freeform®, Rock Hill, USA) (Figure 2). Finally, a curved channel was designed inside the scaphoid 3D model in accordance with the patented design (Figure 3) [15, 16]. The CAD file of the designed prosthesis is finally converted and saved in a standard tessellation language (STL) file format.

### 2.2. FFF PEEK 3D Printer

**Figure 4:**
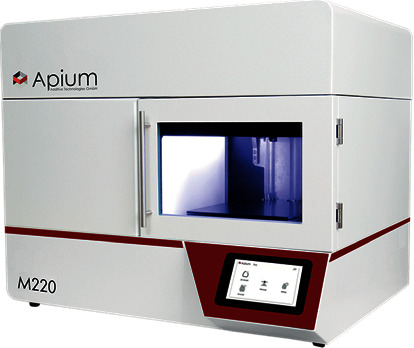
FFF PEEK 3D printer (Apium Additive Technologies GmbH, Karlsruhe, Germany).

The FFF 3D printer used for fabrication of scaphoid prosthesis was an Apium M220, a third-generation desktop printer explicitly designed for PEEK medical additive manufacturing (Apium Additive Technologies GmbH, Karlsruhe, Germany) (Figure 4). It is intended to produce PSIs in a hospital environment according to the biocompatibility standard ISO 10993 [17]. The printer incorporates an advanced temperature management system, which controls the temperature during the printing process in a layer-by-layer fabrication manner. In addition, to prevent contamination, a constant influx of hot airflow is integrated into the printer which filters the atmosphere around the 3D printed part during the fabrication process. The technical specifications of the PEEK FFF 3D printer are listed in Table 1.

### 2.3. PEEK Filament

Due to the physical properties of PEEK biomaterial, FFF 3D printing is a challenge, and it usually requires an iterative process to print the test samples [13, 18]. Therefore, from an economic perspective, the printing feasibility of PEEK scaphoid prosthesis was initially conducted with an industrial-grade 1.75 mm PEEK filament (Apium 4000 natural, Apium Additive Technologies GmbH, Karlsruhe, Germany). Once established, a medical-grade 1.75 mm diameter PEEK filament developed from Vestakeep® i4 G resin (Evonik Vestakeep®i4 G resin, Evonik Industries AG, Essen, Germany) was used for the fabrication of scaphoid prosthesis. This filament is an implant-grade material that meets the ASTM F2026-17 guidelines—Standard specification for Polyetheretherketone (PEEK) Polymers for Surgical Implant Applications [19, 20]. It is a natural-colored, high-viscosity, and high-performance PEEK polymer widely used for long-term implantable medical devices. The material is supplied either directly as a filament on a spool or as cylindrical pellets, which is used for extrusion (FFF) processing technologies to manufacture the PEEK filament. The medical-grade PEEK filament has a density of 1.30 g/cm^3^, a melting temperature of ~340°C, and a glass transition temperature of ~135-155°C. Besides, the material is very tolerant to gamma radiation, stable against hydrolysis, and suitable for autoclave sterilization process.

### 2.4. FFF PEEK 3D Printing Process Parameters

**Figure 5:**
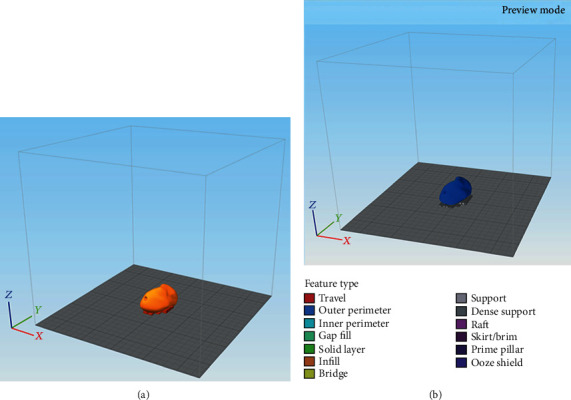
Orientation of the scaphoid prosthesis on the 3D printer's build platform in the 3D slicing software: (a) addition of support structures; (b) g-code generation with selected printing parameters.

The STL file of the scaphoid prosthesis was imported into a commercially available slicing software (Simplify 3D version 4.0, Cincinnati, USA). To prevent collapse and ensure optimal printing, temporary support structures were generated underneath the prosthesis in this software (Figure 5(a)).

Finally, the STL file was digitally sliced with the respective printing parameters to generate a g-code file (Figure 5(b)), which was later on sent to the 3D printer software for printing. The printing parameters used for the light-colored industrial PEEK (Apium PEEK 4000, Apium Additive Technologies GmbH, Karlsruhe, Germany) were similar to the darker medical-grade PEEK filament (Evonik Vestakeep®i4 G resin, Evonik Industries AG, Essen, Germany). The printing parameters selected for the fabrication process are listed in Table 2. To increase the adhesion between the scaphoid prosthesis and the print bed, automatic raft generation functionality integrated into the printer's software was used.

## 3. Results

**Figure 6:**
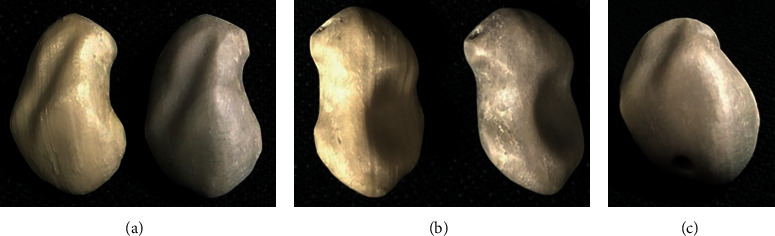
FFF 3D printed scaphoid prosthesis made of industrial-grade (light-colored) and medical-grade (dark-colored) PEEK: (a) radial aspect; (b) ulnar aspect; (c) proximal pole with exit orifice of the channel.

The total printing time for each scaphoid prosthesis was 1 hour and 52 minutes. After printing, the support structures were manually removed, and the scuff marks were trimmed off from the prosthesis. The prints of the prosthesis shown in Figure 6 were not further postprocessed. The scaphoid prosthesis on the left (light-colored) was printed with industrial-grade PEEK filament (Apium 4000), while the prosthesis on the right was printed in medical-grade implantable PEEK biomaterial (Evonik Vestakeep®i4 G resin). No black speck formation or discoloration (improper crystallization) was detected in the test parts. Unlike the industrial-grade 3D printed PEEK scaphoid prosthesis, the surface of the medical-grade PEEK printed version did not display the classical “FFF stair-stepping” phenomenon. Moreover, the articular surfaces and the edges at the channel opening had a smoother finish, which is mandatory to articulate with the cartilage and guide the tendon graft in a frictionless manner.

## 4. Discussion

We report on the first results of a medical-grade 3D printed patient-specific scaphoid prosthesis fabricated at the point-of-care manufacturing. In recent years, material extrusion-based 3D printing of PEEK has achieved a considerable amount of attention for in-house production. The precision of FFF 3D printers has considerably improved and is almost equal to industrial 3D printing technologies for polymers [21]. With the development and availability of medical-grade PEEK filament, it is possible to use FFF 3D printing technology for the production of patient-specific Class III implants for various surgical applications [10]. FFF PEEK 3D printing has certain advantages over other subtractive manufacturing processes, such as milling or injection molding. In a milling process, the amount of waste generation is considerably high. Moreover, the fabrication of complex structures such as the curved channel in the patented scaphoid prosthesis is not possible. The injection molding technique requires less material; however, the technology is more suitable for mass production, and its use in patient-specific or customized implant production is limited [10].

The three major standard organizations that drive the advancement and innovation in medical devices are the ASTM International, the International Standards Organization (ISO), and the Association for the Advancement of Medical Instrumentation (AAMI). These organizations develop consensus technical standards for a wide range of materials, including PEEK [19]. Besides, the Food and Drug Administration (FDA) has recognized some standards for PEEK medical products. It states that by conforming to the abovementioned technical standards, the manufacturer is exempt from the fundamental material property submission reports [20]. In point-of-care manufacturing, maintaining high efficacy and manufacturing quality of the printed parts are of paramount importance and one of the fundamental tasks in compliance with these regulations. Therefore, specific operational and regulatory standards should be established to assess whether the intended 3D printed part conforms to its clinical use. Furthermore, standard organization-based certification for quality management protocols (ISO 13485) including a risk-based approach (ISO 14971) for the whole process, including data conversion, modeling, 3D printing and all post-processes should be integrated into a hospital environment.

PEEK is suitable for orthopedic implants, which are in direct contact with the bone. It is considered as an alternative material in total hip arthroplasty, to avoid metal-metal debris and to minimize the risk of particle-induced aseptic implant loosening [22, 23]. In hand and wrist surgery, PEEK-related complications, such as foreign body synovitis, can occur in a total wrist arthroplasty because of the shearing forces on the implant [24]. Our task as a research lab was to demonstrate and illustrate the possibility of FFF PEEK 3D printing in a hospital environment. The study results show a smoother integration and faster production potential for in-house PEEK PSI manufacturing. Furthermore, as FFF 3D printed parts are anisotropic, appropriate orientation of the scaphoid prosthesis on the 3D printer's build platform concerning its clinical use should be considered. As adhesion is made layer by layer, the printed part will be less weak if the force is applied 90° to the layer and much stronger if the forces are applied along the layer direction, whereas if the center of rotation of the scapholunate axis is oriented perpendicular to the printed layers, the forces of transmission will be in the axial direction. Therefore, we chose the specific orientation of the scaphoid prosthesis for 3D printing [25]. The suspension of the prosthesis is maintained through a fiber-wire augmented tendon graft, which is passed through the curved channel. A rough surface inside the channel could lead to a better connection between the tendon and the PEEK surface. The well-known osseointegration abilities of PEEK into the bone might also contribute to the adhesion between the tendon and the PEEK surface [23, 26–28].

The in-hospital production of PEEK itself by FFF 3D printing is technically demanding and requires a lot of experience especially in the field of FFF 3D printing technology. The printer needs meticulous inspections and maintenance to secure a stable, reliable, and reproducible printing environment to perfectly maintain the print parameters listed in Table 2 during the printing process. If not maintained appropriately, formation of irregularities, color changes, and delamination can potentially develop in the printed parts, which suggest uncontrollable thermodynamically driven changes during the printing process.

Finally, with this proof of concept, further studies regarding the biomechanical properties of the postprocessed patented PEEK scaphoid prosthesis to evaluate the joint cartilage and the channel-tendon graft interface are required. Investigations on the wear properties of PEEK bearing combinations in total knee arthroplasties have shown a cross-shear dependency of PEEK when articulating on hard surfaces such as metal. Therefore, we have the impression that the joint cartilage-PEEK interface depends on the smoothness of the surface of the implant, like in pyrocarbon or titanium implants for carpal bone replacement [29–31]. These types of cartilage damage due to the surface characteristics are underinvestigated and require further evaluation.

## 5. Conclusions

This proof of concept showed the possibility for the additive manufacturing of biocompatible and implantable polymers such as PEEK, in our case, a complex geometry with many joint surfaces in the hospital environment.

## Figures and Tables

**Table 1:** Technical specifications of the FFF PEEK 3D printer.

Parameter	Technical specifications
Number of extruders	1
Nozzle diameter (mm)	0.4
Filament diameter (mm)	1.75
Print volume	132 mm × 132 mm × 120 mm
Temperature management system	Full metal hot end with heating up to 540°C Controlled airflow temperature up to 200°C
Print bed material	316L stainless steel
Machine operation software	Apium control software
Slicing software compatible	Simplify 3D

**Table 2:** Printing parameters selected for FFF 3D printed PEEK scaphoid prosthesis.

*Extruder*	
Nozzle diameter (mm)	0.4
*Temperature*	
Extruder temperature (°C)	485
Airflow temperature (°C)	170
*Layer*	
1st layer height (mm)	0.1
Top solid layers	4
Bottom solid layer	4
Outline/perimeter shells	2
*Infill*	
Internal fill pattern	Rectilinear
External fill pattern	Rectilinear
Interior fill percentage	80%
Raster angle	45/-45
*Support*	
Support infill percentage	40%
Support pillar resolution (mm)	4
*Speed (mm/min)*	
Printing speed	1500

## Data Availability

Availability of the digital STL and g-code files is restricted due to the ownership of the patent by Medartis AG. Requests for a patient-specific scaphoid replacement should be made to the abovementioned company. More data on the material and the printer can be found at https://apiumtec.com.
